# Bioengineered Tricomposite Hydrogel Enhances Chondrogenic Phenotype and Hyaline Matrix Formation in Human Chondrocytes

**DOI:** 10.3390/gels12010035

**Published:** 2025-12-31

**Authors:** Antonio Rojas-Murillo, David Andrés de la Garza-Kalife, Jorge Lara-Arias, Héctor Leija-Gutiérrez, Rodolfo Franco-Márquez, Diana Laura Morales-Wong, Félix Vilchez-Cavazos, Elsa Nancy Garza-Treviño, Mario Simental-Mendía

**Affiliations:** 1 Department of Biochemistry and Molecular Medicine, Universidad Autonoma de Nuevo Leon, Monterrey 64460, Mexico; juan.rojasmrll@uanl.edu.mx (A.R.-M.); david.delagarzaka@uanl.edu.mx (D.A.d.l.G.-K.); elsa.garza.tr@uanl.edu.mx (E.N.G.-T.); 2 Orthopedic Trauma Service, University Hospital “Dr. José Eleuterio González”, School of Medicine, Universidad Autonoma de Nuevo Leon, Monterrey 64460, Mexico; jorge.larars@uanl.edu.mx (J.L.-A.); jose.vilchezcvz@uanl.edu.mx (F.V.-C.); 3 Center for Research in Physical and Mathematical Sciences, Universidad Autonoma de Nuevo Leon, San Nicolás de los Garza 66455, Mexico; hector.leijagt@uanl.edu.mx; 4 Department of Anatomic Pathology and Cytopathology, University Hospital “Dr. José Eleuterio González”, School of Medicine, Universidad Autonoma de Nuevo Leon, Monterrey 64460, Mexico; rodolfo.francomrqz@uanl.edu.mx (R.F.-M.); diana.moraleswng@uanl.edu.mx (D.L.M.-W.)

**Keywords:** articular cartilage, amniotic membrane, fibrin scaffold, tissue engineering, chondrocyte, ECM-derived biomaterials

## Abstract

Fibrin hydrogels are biocompatible but often lack instructive cues needed to sustain chondrocyte phenotype and cartilage-like matrix formation; therefore, we investigated whether a tricomposite fibrin hydrogel incorporating decellularized articular cartilage matrix (dACM) and decellularized amniotic membrane matrix (dAMM) enhances human articular chondrocyte performance in vitro. Human articular chondrocytes were encapsulated in tricomposite or fibrin-only hydrogels and cultured for 28 days, evaluating degradation kinetics, viability and cell density, histological remodeling (H&E, Masson’s trichrome, Safranin O), immunohistochemistry for type II collagen, aggrecan, and type I collagen, and qPCR of SOX9, COL2A1, ACAN, RUNX2, COL1A2, and COL10A1. The tricomposite remained cytocompatible (~99% viability), supported marked cell expansion (~250% by day 28), and degraded more slowly than fibrin controls. It increased chondrogenic gene expression (SOX9 >3-fold vs. control by day 28; sustained COL2A1 at 1.5–2-fold; early ACAN at 3–5-fold) while attenuating off-target transcriptional programs (RUNX2 ~50% of control, reduced COL1A2, and negligible COL10A1). Consistently, histology showed progressive lacuna-like morphology and proteoglycan-rich matrix accumulation, accompanied by strong type II collagen and aggrecan immunoreactivity and reduced type I collagen. Overall, adding dACM and dAMM to fibrin improved hydrogel biofunctionality and promoted hyaline-like extracellular matrix assembly, supporting further evaluation of this cell-instructive platform for focal articular cartilage repair.

## 1. Introduction

Articular cartilage is a highly specialized connective tissue that distributes mechanical loads and provides a smooth, low-friction surface for joint articulation. Its dense extracellular matrix (ECM), composed primarily of type II collagen and aggrecan, confers to the tissue with the viscoelastic and compressive properties necessary for joint function [1,2]. However, its avascular nature, low cell density, and low cell density of resident chondrocytes severely restrict the tissue’s intrinsic capacity for repair following focal injury [3,4]. As a result, untreated defects often enlarge over time and may evolve into degenerative osteoarthritis, highlighting the clinical demand for effective regenerative strategies [5,6,7,8].

Tissue engineering approaches aim to replicate both the biological properties and the structural integrity of native cartilage. Biomaterial-based scaffolds provide a physical framework that supports cellular attachment, proliferation, and ECM production while guiding tissue organization [9]. Among these, fibrin has been widely studied because of its biocompatibility, injectability, and natural role in wound healing [10]. It creates a three-dimensional hydrogel that can encapsulate cells and bioactive molecules, offering a minimally invasive way to fill defects [11,12]. Incorporating decellularized extracellular matrix (dECM) into fibrin hydrogels elevates their biological activity by transforming fibrin from a largely supportive scaffold into a bioinstructive, tissue-mimetic microenvironment [13]. Because dECM retains a diverse repertoire of ECM proteins and bioactive motifs revealed during matrix processing, it provides additional integrin binding and remodeling ligands that strengthen cell adhesion, promote migration, and support cell-mediated matrix deposition and turnover, signals that fibrin alone cannot fully reproduce [14,15]. In parallel, dECM contributes sulfated glycosaminoglycan-rich domains that bind and concentrate soluble factors, acting as an affinity reservoir that sequesters, protects, and presents growth factors to cells, thereby enhancing local signaling and prolonging bioactive factor availability [16]. When incorporated as particles, dECM can better preserve native like microstructural features and distribute these biochemical and structural cues more uniformly throughout the hydrogel, supporting improved cell viability, proliferation, and lineage-specific differentiation [17,18]. However, fibrin alone is limited by its rapid degradation, low mechanical stability, and limited ability to instruct tissue formation, which restricts its use in long-term cartilage regeneration [19,20,21,22].

Recent studies have focused on the functionalization of scaffolds and hydrogels using biologically active components based on dECM to overcome their limitations [23,24]. Decellularized tissues like articular cartilage matrix (dACM) [5,25,26,27,28] and amniotic membrane matrix (dAMM) [5,29,30,31] have shown promise because they retain native ECM constituents and signaling molecules that influence cell behavior. dACM, derived from decellularized articular cartilage, preserves key structural proteins (type II collagen, aggrecan, chondroitin sulfate) and chondrogenic growth factors such as TGF-β and BMPs [32,33]. These elements are vital for maintaining chondrocyte phenotype, encouraging mesenchymal stem cell differentiation, and promoting hyaline-like matrix formation. Similarly, dAMM offers a structurally complex ECM rich in fibronectin, laminin, and glycosaminoglycans, along with anti-inflammatory and immunomodulatory cytokines like IL-10, EGF, and FGF [33,34]. It has been proposed that incorporating dACM and dAMM into fibrin scaffolds could create a multifunctional, bioinspired composite capable of providing both structural support and biologically relevant signals [5]. To date, the combination has not been analyzed in fibrin scaffolds. Such scaffolds could establish a microenvironment conducive to cartilage-specific gene expression, ECM synthesis, and sustain phenotypic stability.

The study aimed to determine whether the addition of decellularized ECM components (dACM and dAMM) enhances the biological functionality of fibrin-based hydrogels’ ability to maintain the chondrogenic phenotype and potential to support the formation of cartilage-like extracellular matrix, like articular cartilage. Human articular chondrocytes were cultured for 28 days in hydrogel and analyzed for histological staining, biochemical quantification of sulfated glycosaminoglycans, and gene expression of chondrogenic markers such as SOX9, COL2A1, ACAN, and RUNX2. Ultimately, this composite approach may provide a promising platform for developing biomaterials capable of promoting stable hyaline cartilage regeneration in focal articular cartilage defects, contributing to the design of more effective, cell-instructive therapies for early-stage joint degeneration.

## 2. Results and Discussion

### 2.1. Tricomposite Hydrogel Degradation Rate Analysis

**Figure 1:**
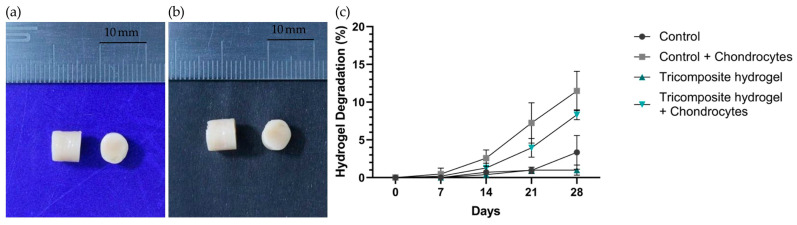
(**a**) Fibrin hydrogel (control); (**b**) Tricomposite hydrogel; (**c**) Hydrogel degradation curve under acellular and cellular conditions.

The resulting hydrogels had a cylindrical shape with standard dimensions of 7.5 mm in height and 6 mm in diameter (Figure 1a,b). The control hydrogel had an average weight of 200 ± 3 mg, whereas the tricomposite hydrogel had a slightly higher weight of 208 ± 5 mg. The degradation of tricomposite and control hydrogels was quantified as the percentage mass loss at days 7, 14, 21, and 28 under cellular and acellular conditions (Figure 1c). In chondrocyte-seeded constructs, both formulations showed gradual mass loss, but the tricomposite hydrogel consistently degraded less than the control. From days 7–14, controls lost ~4–5% of their initial mass, whereas tricomposites lost ~1–2%. Divergence increased thereafter: by day 21, tricomposites had lost 5.2 ± 0.8% versus 8.1 ± 1.5% for controls; by day 28, losses were 7.5 ± 1.3% versus 10 ± 2.7%, respectively. In the absence of cells, tricomposites exhibited minimal erosion (0.5 ± 0.3% by day 28), while acellular controls steadily reached 3.0 ± 0.9%.

The 28-day degradation profile under both cellular and acellular conditions indicates superior stability of the tricomposite hydrogel relative to the control. The lower degradation rate—whether or not chondrocytes were present—suggests greater structural resistance attributable to the incorporation of cartilage and amniotic membrane matrices. This increased stability is advantageous in tissue engineering, providing adequate temporary support for cell maturation and ECM production [35,36]. In particular, the reduced degradation in acellular conditions (0.5 ± 0.3% for the tricomposite versus 3.0 ± 0.9% for the control at day 28) indicates diminished susceptibility to spontaneous hydrolysis and non–cell-mediated proteolytic breakdown [37]. Likewise, in the presence of cells, the slower degradation of the tricomposite (7.5 ± 1.3% versus 10 ± 2.7% at day 28) implies improved interaction with chondrocytes and a possible modulation of local enzymatic activity—factors previously associated with incorporating native matrix components into biomaterials [38,39]. Collectively, these results support the notion that the tricomposite hydrogel provides a more durable microenvironment that favors long-term chondrogenic processes without compromising structural integrity during early differentiation and matrix synthesis.

### 2.2. Cell Viability and Cell Density Analysis

**Figure 2:**
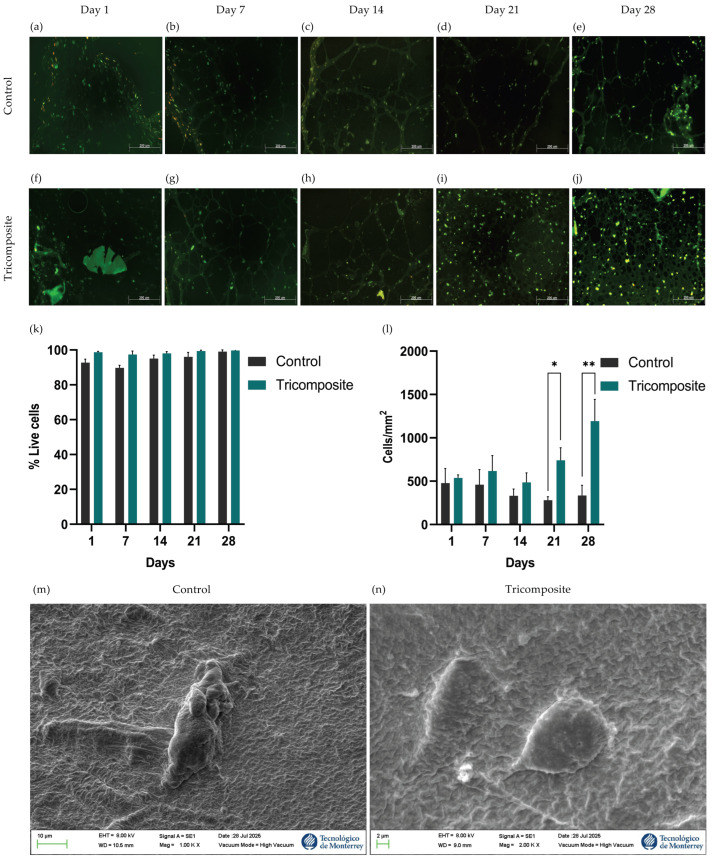
Live/Dead staining at 1, 7, 14, 21, and 28 days in culture for control fibrin hydrogels (**a**–**e**) and tricomposite hydrogels (**f**–**j**); (**k**) Quantification of cell viability; (**l**) Cellularity inside the hydrogels; (**m**,**n**) SEM micrography of control and tricomposite hydrogels, respectively; * *p* < 0.05, ** *p* < 0.01.

Chondrocyte survival and adaptation within control and tricomposite hydrogels were evaluated by live/dead fluorescence staining at days 1, 7, 14, 21, and 28 (Figure 2a–j). Image analysis quantified live (green) and dead (red) cells to calculate viability. In control hydrogels, viability remained ~90 ± 3.2% through day 14 and rose slightly to ~95% between days 14 and 28. Tricomposite hydrogels sustained ~99% viability throughout 28 days of culture, with no statistically significant differences between groups at any time point (Figure 2k).

Qualitative inspection revealed a higher density of live cells in tricomposite hydrogels, which was confirmed by counting cells per square micrometer (cells/µm^2^). On days 1 and 7, controls averaged ~500 cells/µm^2^, whereas tricomposites reached ~650 cells/µm^2^. By day 21, cell density in tricomposites increased to ~800 cells/µm^2^, while controls declined to ~300 cells/µm^2^. At day 28, tricomposites reached ~1200 cells/µm^2^—a ~220% increase over day 1—whereas controls remained near ~350 cells/µm^2^ (Figure 2l). These data indicate that the tricomposite hydrogel not only maintains excellent cytocompatibility and viability but also supports enhanced chondrocyte expansion over time. These results indicate that the tricomposite hydrogel not only maintains excellent cytocompatibility and cell viability but also supports enhanced chondrocyte expansion over time. Cytocompatibility was further confirmed by SEM, which showed chondrocytes attached to the surface of both the control and tricomposite hydrogels (Figure 2m,n).

Results obtained by differential fluorescence staining showed high viability in both groups over 28 days, without statistically significant differences. Nevertheless, the tricomposite hydrogel consistently exceeded 98% viability, remaining ~99% at all time points, indicating a highly cytocompatible milieu, consistent with Xie et al. (2022) using dECM–GelMA hydrogels in human chondrocytes [40]. This high cytocompatibility likely reflects bioactive components preserved in dACM and dAMM, which modulate cellular responses; Zeng et al. (2022) reported a more stable chondrocyte response from day 14 with dECM, while Cao et al. (2021) observed increased survival/phenotype-maintenance genes with amniotic-derived factors through day 28 [25,31].

Quantitative image analysis confirmed a progressive, sustained rise in cell density within the tricomposite hydrogel, with a ~220% increase by day 28 versus controls, indicating that the functionalized hydrogel supports survival and actively stimulates proliferation under 3D conditions—aligning with Mao et al. (2019) (dECM-driven expansion, phenotype integrity ≥21 days) and Bhattacharjee et al. (2020) (amniotic membrane in hydrogels lowers apoptosis and increases density) [37,41]. The decline in control cell density from day 14 may reflect the limited capacity of pure fibrin hydrogel to sustain late-stage proliferation, likely due to greater structural degradation and lack of specific bioactive cues [42]. Overall, the tricomposite hydrogel not only maintains viability but also creates a dynamic microenvironment that promotes chondrocyte expansion and organization, prerequisites for effective cartilage regeneration.

### 2.3. Chondrogenic Markers Gene Expression Analysis

The functionality of the tricomposite hydrogel was evaluated by measuring the expression of key lineage and matrix markers at days 7, 14, 21, and 28. Chondrogenic commitment was assessed via SOX9; hyaline cartilage matrix production was monitored through COL2A1 and ACAN; fibrous cartilage formation and hypertrophic drift were tracked with COL1A2 and COL10A1, respectively; and osteogenic potential was gauged by RUNX2.

**Figure 3:**
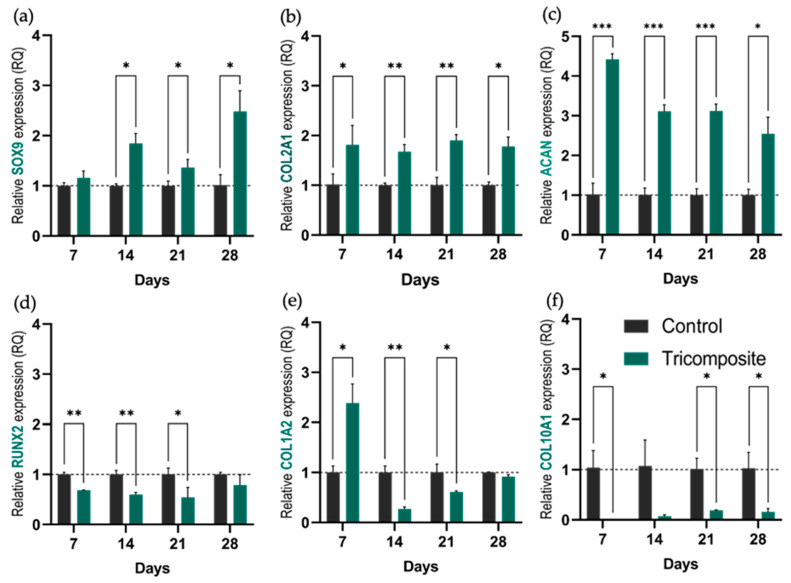
Relative expression of genes associated with the chondrogenic phenotype. (**a**–**d**) Relative expression of SOX9, COL2A1, ACAN, and RUNX2, respectively, in control hydrogels and tricomposite hydrogels at days 7, 14, 21, and 28 of culture; (**e**,**f**) Expression of COL1A2 and COL10A1 as markers of fibrocartilage and hypertrophy. * *p* < 0.05, ** *p* < 0.01, *** *p* < 0.001.

SOX9 expression in tricomposite hydrogels increased continuously over time (Figure 3a). By day 21, levels were approximately twice those of control hydrogels, and by day 28, they exceeded controls by more than threefold. In contrast, control hydrogels maintained low, stable SOX9 expression throughout the culture period, suggesting that the tricomposite hydrogel progressively activates the chondrogenic program, particularly at later stages.

SOX9 showed a progressive increase from day 21 onward, reaching more than three times the control by day 28. This indicates sustained activation and intensification of the chondrogenic lineage, attributable to bioactive signals present in the articular cartilage matrix and the amniotic membrane. Although previous studies such as Yeung et al. (2019) and Dufour et al. (2021) reported increases in SOX9 in chondrogenic hydrogels [43,44], those rises are generally more modest (about 1.5 to 2-fold versus control), highlighting the higher efficacy observed here. This activation aligns with the role of SOX9 as a master regulator of chondrogenesis, initiating the chondrogenic transcriptional program and inhibiting alternative pathways such as osteogenic differentiation regulated by RUNX2 [45].

COL2A1 remained consistently elevated in tricomposite hydrogels, about 1.5 to 2 times higher than controls at every time point (Figure 3b). This sustained expression indicates robust and maintained hyaline matrix synthesis.

The maintenance of high COL2A1 levels throughout culture in the tricomposite hydrogels reinforces this strategy’s capacity to preserve the chondrogenic phenotype of human chondrocytes. Unlike the control group, where COL2A1 remained consistently low, chondrocytes within the functionalized hydrogel sustained expression of this primary structural component of hyaline cartilage. This contrasts with Liang et al. (2017) and Rikkers et al. (2020), who observed progressive decreases in COL2A1 in 3D cultures when specific bioactive signals were absent [46,47], and with Witt et al. (2017), who documented a significant loss of chondrogenic lineage without proper stimuli [48]. These results suggest that the synergistic inclusion of dACM and dAMM within the tricomposite hydrogel provides a more competent environment to maintain key gene expression and prevent the dedifferentiation typical of suboptimal culture conditions.

ACAN showed a pronounced early upregulation in the tricomposite hydrogel (Figure 3c). At day 7, expression was approximately fivefold higher than in controls. Although this fold difference decreased slightly over time, ACAN levels in tricomposites remained at least three times greater than in controls through day 28, reflecting early and persistent proteoglycan deposition.

The elevated ACAN expression from the early days of culture indicates timely activation of biosynthetic processes associated with cartilage matrix formation. ACAN encodes aggrecan, which is associated with high anabolic activity in functional chondrocytes and with preservation of the biomechanical properties of hyaline cartilage [49,50,51]. In contrast to other models where ACAN declines markedly after the first week, here it remained high through day 28 with only a slight progressive reduction, suggesting that the tricomposite hydrogel induces an early anabolic response and sustains it over time, providing a bioactive environment conducive to regenerating matrix similar to native hyaline cartilage.

RUNX2 was consistently higher in control hydrogels. Tricomposite hydrogels exhibited about 50% lower expression with no significant temporal changes (Figure 3d), indicating partial suppression of off-target osteogenesis. The markedly low expression of RUNX2 in the tricomposite group compared to controls suggests partial but sustained inhibition of the osteogenic pathway.

This result is desirable, since RUNX2 activation has been associated with transitions toward osteoblastic differentiation and with the risk of endochondral ossification, which is undesired in cartilage regeneration strategies [52,53]. Several studies have shown that RUNX2 repression supports chondrogenic phenotype stability and prevents hypertrophic or mineralizing changes [45,54]. In this context, the present data reinforce the idea that the tricomposite hydrogel not only promotes key anabolic markers but also helps suppress undesired differentiation pathways.

COL1A2 expression stayed low in both groups (Figure 3e). Tricomposite hydrogels showed a decrease in COL1A2 expression starting on day 14, and although it slightly increased by day 28, it did not surpass the expression level of the control group, suggesting that the tricomposite hydrogel limits fibrous cartilage formation.

COL1A2, a marker associated with fibrous cartilage formation, remained low in both groups throughout the culture period. However, the slight increase observed in controls at day 14 may indicate an undesired phenotypic shift, possibly related to suboptimal culture conditions or lack of specific regulatory signals. This behavior has been described in the absence of adequate inhibitory stimuli, where chondrocytes tend to express genes linked to fibrotic phenotypes [55]. In contrast, the hydrogel functionalized with articular cartilage matrix and amniotic membrane not only stimulated chondrogenic markers but also appeared to exert a selective downregulatory effect on genes associated with non-hyaline tissues. This dual action, promotion of chondrogenesis together with inhibition of fibrotic pathways, is essential for guiding specific regeneration of articular cartilage, as emphasized by Armiento et al. (2019) in repair models where phenotypic specificity is key for long-term success [56].

COL10A1 remained nearly undetectable in tricomposite hydrogels (Figure 3f). The expression in this group hovered near zero at all time points, confirming that neither hydrogel induced hypertrophic drift and that chondrogenic differentiation remained stable.

COL10A1 expression in our hydrogels was virtually undetectable throughout the 28-day culture period. Since COL10A1 marks chondrocyte hypertrophy, its absence suggests that pathways leading to mineralization or terminal hypertrophy were not activated, events typically associated with uncontrolled differentiation [57,58]. This finding is relevant because hypertrophy remains a major challenge in cartilage engineering, and its suppression is a key criterion for validating the stability of the chondrogenic phenotype induced by a biomaterial [59,60].

The gene expression results demonstrate that the tricomposite hydrogel fulfills a dual role: it actively stimulates the chondrogenic pathway while simultaneously repressing osteogenic and other undesired pathways, a fundamental feature for developing functional cartilage tissue. These effects are consistent with reported benefits of using cartilage- and amniotic membrane–derived matrices as bioactive components in tissue engineering [25,38,61].

### 2.4. Production of ECM Components Analysis (IHC and H-Score Evaluation)

**Figure 4:**
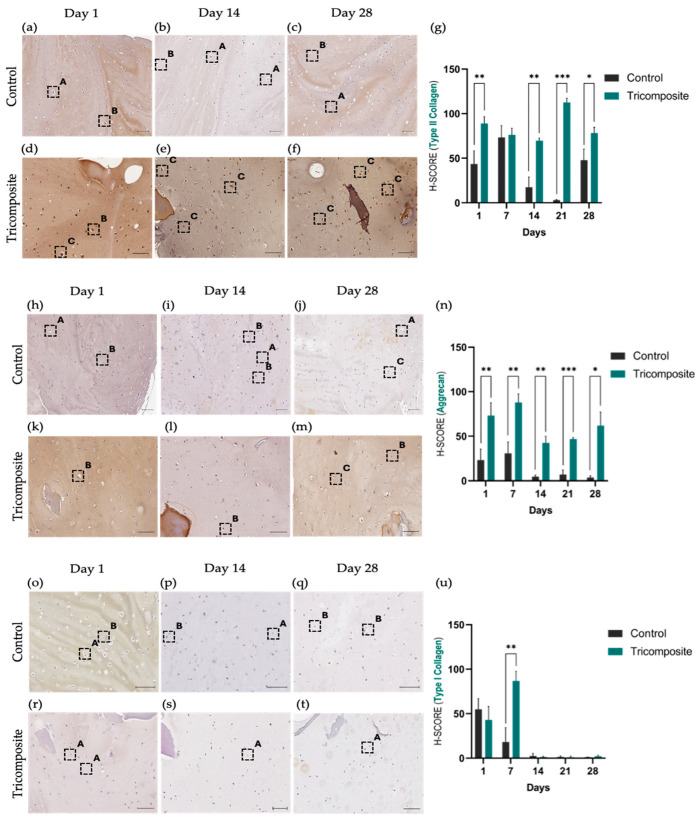
Immunohistochemical evaluation of type II collagen, aggrecan, and type I collagen in control hydrogels and tricomposite hydrogels. (**a**–**g**) Type II collagen: (**a**–**c**) control; (**d**–**f**) tricomposite; (**g**) H-score. (**h**–**n**) Aggrecan: (**h**–**j**) control; (**k**–**m**) tricomposite; (**n**) H-score. (**o**–**u**) Type I collagen: (**o**–**q**) control; (**r**–**t**) tricomposite; (**u**) H-score. Greater staining intensity is observed in tricomposite hydrogels, particularly at day 28. Boxes are labeled as (A) weak, (B) moderate, and (C) strong staining for their respective antibody; *t*-test *p* values: * = *p* < 0.05, ** = *p* < 0.01, *** = *p* < 0.001; scale bar = 20 µm.

In control hydrogels, immunohistochemistry for type II collagen showed a low and essentially constant signal at days 1, 14, and 28, with predominantly faint cytoplasmic staining and scarce deposition in the immediate matrix (Figure 4a–c). In the tricomposite hydrogel, the signal increased progressively over time: mild and focal at day 1, marked at day 14 with evident deposition in the pericellular and adjacent matrix, and intense and more homogeneous at day 28, detectable both in the cytoplasm and ECM (Figure 4d–f). The H-score mirrored this temporal increase and confirmed a significant advantage of the tricomposite hydrogel over the control at day 28 (Figure 4g). Spatial distribution was more defined in regions containing dACM, suggesting a microenvironmental effect of the native matrix on type II collagen synthesis and organization. The validation of the tricomposite hydrogel at the protein level via immunohistochemistry revealed differential and time-regulated expression of key extracellular matrix (ECM) components of hyaline cartilage. In particular, type II collagen immunoreactivity was robust and progressively increased in cultures on the tricomposite hydrogel, with both intracellular and extracellular matrix staining. Expression visibly increased from day 14 and peaked at day 28, indicating not only active collagen II synthesis but also proper secretion and extracellular organization. In contrast, control hydrogels exhibited weak staining limited to a few cells, with no clear evidence of matrix deposition, suggesting poor chondrogenic induction in the absence of bioactive cues.

These results partially align with those reported by Goldberg-Bockhorn et al. (2022), who used decellularized porcine cartilage matrices to stimulate human chondrocytes [62]. In their studies, collagen II expression increased from day 14, although extracellular deposition was less evident than in our work. Similarly, Cheng et al. (2011) and Lin et al. (2020) reported that decellularized matrices in 3D systems promote type II collagen accumulation between days 21 and 28 [63,64], which closely matches the kinetics observed here. However, unlike those studies, the tricomposite hydrogel exhibited a homogeneous and intense signal from earlier stages, suggesting that the combination of dACM and dAMM creates a more effective microenvironment for collagen II production and organization in human chondrocytes.

Controls showed a weak signal of aggrecan with no net changes throughout the culture period. In the tricomposite hydrogel, aggrecan increased from day 1 and continued to rise at day 14 (Figure 4h–j), with pericellular accumulation and zones of matrix enrichment around cell clusters. By day 28, immunohistochemistry showed an intense and widely distributed signal across the hydrogel (Figure 4k–m), with H-score values higher than controls at all time points, the difference being greatest at day 28 (Figure 4n). Again, dACM-rich areas exhibited higher signal density, consistent with the localized release/spatial presentation of chondrogenic cues by the native matrix. Aggrecan staining followed a time-dependent increase in cultures on the tricomposite hydrogel, showing intense and homogeneous matrix accumulation by day 28, in contrast to the weak, cytoplasmic signal in controls.

Given aggrecan’s role in cartilage biomechanical integrity and load-bearing function [50], its abundance in the tricomposite hydrogel group suggests functional activation of the chondrogenic phenotype. Recent studies have highlighted the importance of promoting proteoglycan synthesis to achieve effective cartilage regeneration, especially in 3D culture settings [65,66].

In the control hydrogel, type I collagen showed decreased expression since day 1 (Figure 4o–q). In the tricomposite hydrogel, the signal was similar or slightly lower at day 1, decreased gradually by day 14, and was markedly low or nearly undetectable by day 28 (Figure 4r–t). The H-score showed a clear time-dependent decrease in the tricomposite hydrogel, in contrast to the maintenance observed in the control (Figure 4u).

Taken together, these data indicate that the tricomposite hydrogel promotes a chondrogenic program, increasing the production of type II collagen and aggrecan, and discourages fibrocartilaginous features, decreasing the type I collagen presence during three-dimensional culture.

Type I collagen expression, a marker of fibrous cartilage, significantly decreased in cultures with the tricomposite hydrogel, indicating suppression of undesired pathways associated with a fibrocartilaginous phenotype. While expression in the control group remained stable and moderate, it was reduced by over 70% between days 1 and 28 in the tricomposite group. This decrease reflects the hydrogel’s ability to restrict differentiation toward mechanically inferior tissues, a phenomenon also seen in models incorporating inhibitory signals or hyaline cartilage–mimicking matrices [67,68].

Semi-quantitative scoring (H-score) confirmed these microscopic findings, revealing significant differences in favor of the tricomposite hydrogel in hyaline cartilage markers (type II collagen and aggrecan) and a progressive reduction in type I collagen expression. Compared to other hydrogel systems, the H-score values for type II collagen in the tricomposite group exceed those reported for pure fibrin [43] or synthetic biomaterials [69] lacking native ECM components. This behavior may be attributed to the bioactive nature of the tricomposite hydrogel, which mimics structural and biochemical signals of cartilage, facilitating ordered ECM synthesis and phenotypic maturation of chondrocytes. Together, these findings reinforce the idea that the tricomposite hydrogel not only supports the transcriptional activation of chondrogenic pathways but also translates this activation into the effective and organized production of essential hyaline cartilage components. Simultaneously, it limits type I collagen synthesis, indicating a more stable and tissue-specific phenotype.

### 2.5. Histological Characterization (H&E, Masson Trichrome and Safranin O/Fast Green)

**Figure 5:**
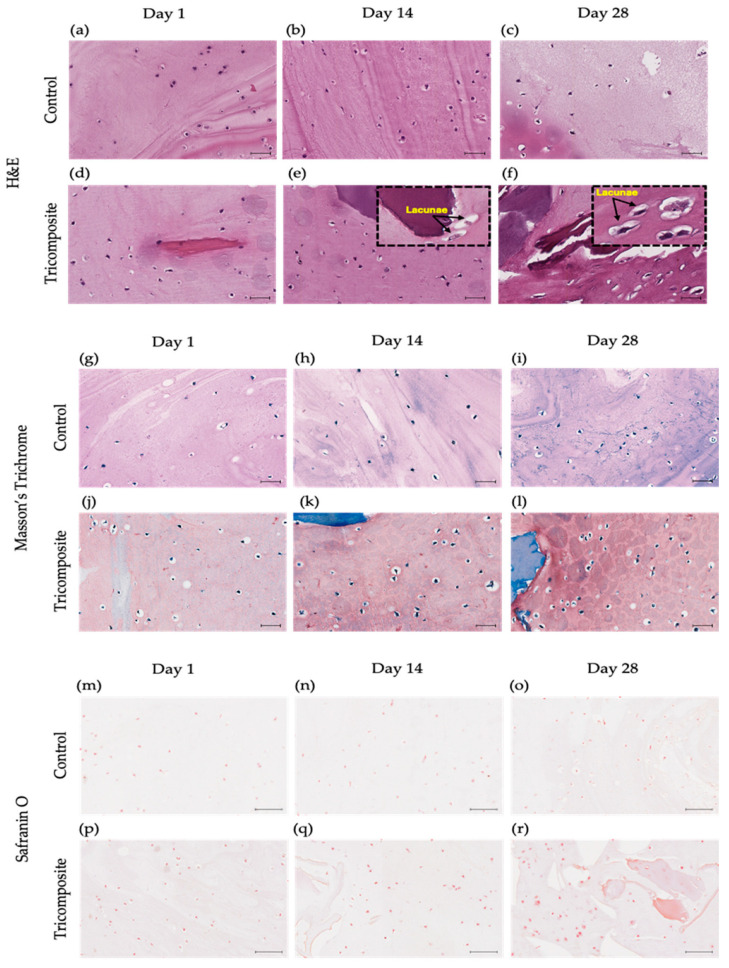
Histology of control hydrogels and tricomposite hydrogels at days 1, 14, and 28. (**a**–**f**) H&E: (**a**–**c**) control; (**d**–**f**) tricomposite. (**g**–**l**) Masson’s Trichrome: (**g**–**i**) control; (**j**–**l**) tricomposite. (**m**–**r**) Safranin O/Fast Green: (**m**–**o**) control; (**p**–**r**) tricomposite; scale bar = 20 µm.

In control hydrogels, cells remained dispersed, rounded, and without lacunar organization across all time points, with a tenuous matrix and no clear pericellular niche (Figure 5a–c). In the tricomposite hydrogel, cells were closer together and embedded in a homogeneous matrix from day 1; by day 14, clusters with a clear halo appeared (incipient lacunae); and by day 28, multiple well-defined lacunae containing viable chondrocytes were observed—features consistent with hyaline cartilage (Figure 5d–f).

In fibrin-only controls, the blue signal of the Masson trichrome stain was faint at day 1, remained weak and discontinuous at day 14, and by day 28 was still limited, with minimal pericellular or cytoplasmic uptake (Figure 5g–i). In contrast, tricomposite hydrogels (fibrin + dACM + dAMM) showed an early blue signal at day 7 that intensified by day 14, spreading from the extracellular matrix into the pericellular zone and visibly tinting the cell cytoplasm. By day 28, the blue coloration was intense and widespread, revealing collagen production by the cells (Figure 5j–l).

Safranin O/Fast Green staining was weak or absent in controls at all time points, with no evidence of a high content of proteoglycan-rich matrix (Figure 5m–o). In the tricomposite hydrogel, the signal appeared early and intensified over time, reaching strong pericellular and extracellular staining by day 28 (Figure 5m–r). The signal was more intense in dACM and dAMM-rich regions, suggesting local GAG synthesis/retention favored by components of the decellularized matrix. The co-occurrence of intense Safranin O/Fast Green staining, well-defined lacunae, the IHC and gene expression profile (↑ COL2A1/ACAN, ↓ COL1A1) supports chondrogenic phenotypic maturation in the tricomposite hydrogel.

Control hydrogels showed spherical, scattered cells without hyaline-like structures, consistent with 3D systems lacking structural/biochemical cues for reorganization [69,70]. In contrast, tricomposite hydrogels displayed progressive lacuna formation from day 14, indicating a microenvironment that supports cell–matrix and cell–cell interactions required to mimic native cartilage architecture.

The homogeneous matrices and pericellular halos in Safranin O–stained sections indicate active proteoglycan synthesis and GAG-rich ECM—key components of hyaline cartilage [71]—and align with the notion that biomaterials emulating cartilage stiffness, porosity, and biochemical composition facilitate structural reconstitution [72]. Consistently, the weak Safranin O in controls suggests limited proteoglycan synthesis in materials without specific bioactive signals [69].

In tricomposite hydrogels, strong and progressively intensifying Safranin O—especially around dACM—suggests phenotype activation via direct ECM interaction; the robust intracellular/pericellular signal indicates active GAG biosynthesis essential for functional matrix assembly [73]. GAG accumulation underpins biomechanical properties and chondrocyte function; Safranin O detection is a key, often highly sensitive, marker of chondrogenic maturation [74] and matches reports using natural matrices or hydrogels enriched with chondrogenic cues [75,76]. The predominant localization in dACM-containing regions supports the native matrix as a reservoir of inductive cues via controlled protein release or spatial presentation [77], with cell–matrix feedback consolidating the chondrogenic phenotype in 3D biomimetic platforms [78].

Prior work with decellularized cartilage- or amnion-enriched hydrogels shows the promotion of viability/differentiation and remodeling that favors lacunae and isogenous groups over extended culture [79,80]. The present behavior is consistent, suggesting that combining fibrin, cartilage matrix, and amniotic membrane delivers mechanical and chemical signals that drive condensation and domain organization [31,61].

Overall, the structural evolution—lacunae formation together with proteoglycan-rich ECM—suggests functional coupling between ECM production and architecture, reflecting maturation akin to cartilage development [81,82]. These findings indicate that the tricomposite hydrogel not only provides a biocompatible environment but also enables cellular reorganization and organized cartilage-specific ECM production, outperforming controls and underscoring the importance of bioactive native-tissue components for structural and functional regeneration consistent with articular cartilage physiology [25,61].

The proposed fibrin + dACM + dAMM tricomposite may support cell-instructive therapies for early-stage joint degeneration by acting as an injectable, ECM-enriched microenvironment that provides cartilage-specific cues, helping maintain chondrocyte phenotype and promote hyaline-like matrix formation in focal defects. In our in vitro model, it showed good cytocompatibility and cell expansion, increased chondrogenic markers (SOX9, COL2A1, ACAN), and reduced fibrocartilaginous/hypertrophic markers (RUNX2, COL1A2, COL10A1), consistent with proteoglycan-rich deposition and type II collagen/aggrecan staining. These findings suggest a local instructive effect relevant to early degeneration, but the therapeutic extent still requires in vivo validation (integration, function in inflammatory/degenerative settings, durability under loading, and safety).

## 3. Conclusions

The fibrin-based tricomposite hydrogel (fibrin + dACM + dAMM) was cytocompatible, sustaining chondrocyte viability from day 1 and reaching ~250% proliferation by day 28. Over the 28-day culture, it preserved a chondrogenic program—evidenced by sustained SOX9 and COL2A1—and reversed the fibrocartilage marker COL1A2 from day 14, indicating phenotypic redirection toward hyaline cartilage. The three-dimensional microenvironment promoted lacunar organization typical of native cartilage and supported deposition of cartilage-specific extracellular matrix within the hydrogel, as corroborated by histology and matrix-level readouts (including sGAG quantification). Collectively, these findings indicate that embedding decellularized ECM components enhances the biological functionality of fibrin hydrogels and helps overcome limitations of simple matrices, positioning this tricomposite hydrogel as a promising, cell-instructive platform for stable hyaline-like cartilage regeneration in focal articular defects.

## 4. Materials and Methods

### 4.1. Preparation of Decellularized Articular Cartilage and Amniotic Membrane Matrices

The dACM was prepared as previously reported [5]. In detail, full-thickness human articular cartilage (2 mm) was aseptically harvested from femoral condyles, patella and trochlear groove of twenty cadaveric donors (As part of the project: Design of a fibrin-based scaffold incorporating biological matrices with chondrogenic potential, approved by the Ethics Committee, approval number BI23-001), rinsed three times in PBS (1×, Gibco, Grand Island, NY, USA) containing 1% Antibiotic–Antimycotic (100×, 10,000 units/mL of penicillin, 10,000 µg/mL of streptomycin, streptomycin, and 25 µg/mL of Amphotericin B, Gibco, USA), then stored at –80 °C. Approximately 205 g of tissue per batch underwent five cryoshock cycles (liquid nitrogen, 5 min; thaw in PBS, 10 min), and crushed with a blender. Particles were incubated at 4 °C for 24 h in hypotonic buffer (10 mM Tris–HCl, pH 8.0; 2 mM EDTA; 100 mM KCl; 5 mM MgCl_2_), then in the same buffer with 0.5% SDS for 18 h and washed for 36 h with buffer changes every 12 h. After three distilled water rinses and three PBS washes to eliminate the remaining detergents, material was frozen at –80 °C, lyophilized (24 h), and milled to fine powder using a SPEX Freezer/Mill 6870 (SPEX SamplePrep, Metuchen, NJ, USA) and a Micron K10 pulverizer (Micron, Shanghai, China). dACM was sterilized by ethylene oxide and stored at –80 °C.

The dAMM was prepared from thirty human placentas collected post-elective cesarean (As part of the project: Design of a fibrin-based scaffold incorporating biological matrices with chondrogenic potential, approved by the Ethics Committee, approval number BI23-001). Amniotic membranes were separated in a sterile environment, rinsed thrice in cold PBS with Antibiotic–Antimycotic, cut into ~1 cm^2^ fragments, and frozen at –80 °C. Following 125 g of tissue underwent five cryoshock cycles (liquid nitrogen, 5 min; thaw in PBS, 10 min), then 4 h in 0.1% Tween 80 (Sigma-Aldrich, St. Louis, MO, USA), 1 h in 0.1 M NaOH (7%, JALMEX, Guadalajara, Mexico), and two sequential 1 h treatments with 0.15% peracetic acid (PAA, 15%, Cetik, Cualiacán, Mexico) in 96% ethanol (96%, CTR, Monterrey, Mexico), interspersed with 0.1 M NaOH. Samples were rinsed in 70% ethanol for 1 h, washed in PBS for 2h (3 times), frozen, lyophilized (24 h), and milled as above. dAMM was sterilized by ethylene oxide and stored at –80 °C.

For hydrogel preparation, both matrices (dACM and dAMM) were sieved to obtain particles in the 75–250 µm size range prior to the sterilization step.

### 4.2. Tricomposite Scaffold Preparation

Tricomposite scaffolds were fabricated by combining a commercial fibrin sealant with dACM and dAMM. First, 220 mg of fibrinogen powder (Tisseel, Baxter, Vienna, Austria) was dissolved in 1 mL of aprotinin solution (1500 IU/mL) at 37 °C under agitation until fully soluble. Human articular chondrocytes (1 × 10^6^ cells), suspended in 50 µL of PBS, were then added to the fibrinogen and aprotinin solution and mixed under gentle agitation (90 rpm in a sterile glass vial of 5 mL) to avoid harming the cells. After obtaining a homogeneous suspension, 1.5 mg of dACM and 6 mg of dAMM were incorporated and gently stirred to ensure even distribution of the matrices. Those amounts were determined in our previous work [5]. Briefly, chondrocytes were exposed to different concentrations of soluble extracts from dACM and dAMM. Cytotoxicity and proliferation assays were then performed, followed by analysis of chondrogenic gene expression. The concentrations selected for the hydrogel preparation were those that produced the highest cell proliferation and the greatest upregulation of chondrogenic markers.

In parallel, 250 IU of thrombin was reconstituted in 1 mL of calcium chloride solution (0.44 µmol CaCl_2_) at 37 °C. The fibrinogen, cell and matrix suspension and the thrombin and calcium chloride solution were loaded into the two chambers of a dual-syringe system and co-extruded at a 1:1 volume ratio into custom molds (final volume 200 µL). Constructs were allowed to polymerize at 37 °C until gelation was complete and washed with PBS.

### 4.3. Scaffold Degradation Analysis

Degradation of hydrogels was evaluated for four experimental groups: fibrin scaffold, fibrin scaffold seeded with chondrocytes, tricomposite scaffold and tricomposite scaffold seeded with chondrocytes. Triplicate samples of each group were freeze-dried and weighed to determine the initial dry weight (W_i_).

Same mass fresh scaffolds were then transferred to 24-well plates and incubated in 1.5 mL of DMEM/F12 supplemented with 10% bovine fetal serum at 37 °C in a humidified atmosphere with 5% CO_2_. At 7, 14, 21 and 28 days, scaffolds were gently rinsed with deionized water, freeze-dried and weighed to obtain the residual dry weight (W_t_). Degradation was calculated as a percentage mass loss according to the equation below:

$$Degradation\;\left(\%\right)=\frac{W_{i}-W_{t}}{W_{i}}\times 100$$

### 4.4. In Vitro Cytocompatibility, Cell Viability and Cellularity Analysis

In vitro cytocompatibility, cell viability and cellularity were evaluated for fibrin (control) and tricomposite scaffolds at 1, 7, 14, 21 and 28 days. At each time point, scaffolds were incubated with the LIVE/DEAD™ Viability/Cytotoxicity Kit (Invitrogen, Carlsbad, CA, USA) following the manufacturer’s protocol as adapted for three-dimensional constructs. Briefly, 2 µL of ethidium homodimer-1 (Homodimer D1) stock solution was diluted in 5 mL of PBS, then 2.5 µL of Calcein AM was added. Scaffolds were submerged in this staining solution for 10 min at room temperature in the dark. After staining, samples were cryosectioned at 8 µm thickness using a cryostat. Sections were mounted with aqueous mounting medium and imaged by fluorescence microscopy to capture live cells (green) and dead cells (red).

Quantitative analysis was performed using ZEN Blue software (Carl Zeiss Microscopy GmbH, Göttingen, Germany). For each scaffold, three independent samples were analyzed; from each sample, ten cryosections were prepared, and on each section, ten random fields were imaged. Total cell number was determined by counting green and red fluorescent signals. Cell viability was calculated as the percentage of live cells relative to the total cell count. Cellularity was expressed as the absolute number of live cells per field.

### 4.5. Chondrogenic Gene Expression Analysis

Chondrogenic gene expression was evaluated in fibrin and tricomposite scaffolds seeded with human articular chondrocytes. Constructs were prepared as described and maintained in DMEM/F12 with 10% FBS. At 7, 14, 21 and 28 days, three scaffolds per group were collected and immediately immersed in RNAlater (Invitrogen, Carlsbad, CA, USA) to stabilize RNA. Prior to extraction, samples were removed from RNAlater, briefly rinsed with cold PBS to remove excess reagent, and then pulverized under chilled conditions. Total RNA was extracted using the RNeasy Mini Kit (Qiagen, Germantown, MD, USA) following the manufacturer’s protocol, including on-column DNase digestion. RNA concentration and purity were assessed by spectrophotometry (A260/A280 ratio).

First-strand cDNA was synthesized from 500 ng of total RNA using the SuperScript III First-Strand Synthesis System (Invitrogen, Carlsbad, CA, USA) according to the supplier’s instructions. Quantitative PCR was performed on a 7500 Real-Time PCR System (Applied Biosystems, Waltham, MA, USA) using TaqMan Gene Expression Assays for B2M (Hs99999907_m1), ACAN (Hs00153936_m1), SOX9 (Hs00165814_m1), COL2A1 (Hs00156568_m1) and RUNX2 (Hs01047973_m1). Each 20 µL reaction contained 10 µL of TaqMan Universal PCR Master Mix, 1 µL of the appropriate probe mix and 2 µL of diluted cDNA. All samples were run in triplicate alongside no-template controls. Data were analyzed by the 2^–ΔΔCt^ method, normalizing each target gene to B2M and expressing results relative to the fibrin control of each time point.

### 4.6. Histological and Immunohistochemical Analysis

Histological and immunohistochemical analyses were performed on fibrin and tricomposite scaffolds seeded with chondrocytes, at 7, 14, 21 and 28 days of culture. At each time point, three scaffolds per group were rinsed briefly in PBS, fixed in 4% paraformaldehyde for 24 h at 4 °C, then dehydrated through an ethanol series and embedded in paraffin by standard methods. From each block, 6 µm sections were cut on a microtome and mounted on glass slides.

For general morphology and nuclear evaluation, sections were stained with DAPI (4′,6-diamidino-2-phenylindole; Vector Laboratories) to detect residual DNA or nuclear material, then with hematoxylin and eosin to assess tissue organization and cell distribution. Proteoglycan content was visualized by Safranin O staining (Sigma-Aldrich, St. Louis, MO, USA) and overall collagen architecture by Masson’s trichrome staining (Sigma-Aldrich, St. Louis, MO, USA). Slides were dehydrated, cleared and coverslipped before imaging.

To assess extracellular matrix production, immunohistochemistry was performed for hyaline cartilage markers—type II collagen and aggrecan—and for the fibrocartilage marker type I collagen. Sections were deparaffinized, rehydrated and subjected to antigen retrieval in citrate buffer (pH 6.0) at 95 °C for 20 min. Endogenous peroxidase activity was quenched with 0.3% hydrogen peroxide in methanol for 30 min. After blocking in 5% bovine serum albumin for 1 h at room temperature, slides were incubated overnight at 4 °C with one of the following primary antibodies: anti–collagen II (1:400; ab34712, Abcam, Cambridge, UK), anti–aggrecan (1:100; ab3778, Abcam, Cambridge, UK) or anti–collagen I (1:200; ab34710, Abcam, Cambridge, UK). Detection was carried out using an HRP/DAB kit (ab64264, Abcam, Cambridge, UK) following the manufacturer’s instructions and counterstained with Gill’s hematoxylin. Negative controls omitted the primary antibody.

All stained sections were imaged on an Olympus AX70 microscope under identical exposure settings. For each scaffold, three slides were analyzed and ten random fields per slide were captured. Positive staining was quantified using ImageJ (version 1.54g; National Institutes of Health [NIH], Bethesda, MD, USA) by thresholding the DAB signal and dividing by total tissue area. In addition, an H-score was calculated for each marker to combine staining intensity and frequency. For each field, cells were classified by intensity (1 = weak, 2 = moderate, 3 = strong) and the percentage of cells in each category (P_1_–P_3_) was recorded. The H-score was calculated as:

$$H-score=\;\left(1\times P_{1}\right)+\left(2\times P_{2}\right)+\left(3\times P_{3}\right)$$

where P_i_ is the percentage of cells staining at intensity i. H-scores range from 0 (no staining) to 300 (100% of cells strongly positive). Results are reported as mean ± SD for each group and time point.

### 4.7. Statistical Analysis

Statistical analyses were performed on data from three independent experiments, each with triplicate samples. All analyses were carried out in GraphPad Prism 9.0.2 (GraphPad Software, La Jolla, CA, USA). Quantitative variables such as scaffold degradation, cell viability, cellularity, immunohistochemical positive area and relative gene expression were first assessed for normality using the Shapiro–Wilk test. For comparisons involving two groups at a single time point, an unpaired t-test with Welch’s correction was applied. For analyses involving multiple groups and time points, two-way analysis of variance (ANOVA) was used, followed by Tukey’s multiple comparisons test. Data are reported as mean ± standard deviation. Statistical significance was defined as *p* < 0.05 (*), *p* < 0.01 (**) and *p* < 0.001 (***).

## Data Availability

The original contributions presented in this study are included in the article. Further inquiries can be directed to the corresponding author.

## Abbreviations

ACAN	Aggrecan (gene)
B2M	beta-2-microglobulin (gene)
BMPs	Bone morphogenetic proteins
COL1A2	Collagen type I alpha 2 chain (gene)
COL2A1	Collagen type II alpha 1 chain (gene)
COL10A1	Collagen type X alpha 1 chain (gene)
dACM	decellularized articular cartilage matrix
dAMM	decellularized amniotic membrane matrix
dECM	decellularized extracellular matrix
ECM	Extracellular matrix
EGF	Epidermal growth factor
FGF	Fibroblast growth factor
GAG	Glycosaminoglycan
GelMA	Gelatin methacrylate
IHC	Immunohistochemistry
IL-10	Interleukin-10
RUNX2	Runt-related transcription factor 2 (gene)
sGAG	Sulfated glycosaminoglycans
SOX9	SRY-box transcription factor 9 (gene)
TGF-β	Transforming growth factor beta
